# A Risk-Scoring Model to Predict One-year Major Adverse Cardiac Events after Percutaneous Coronary Intervention

**Published:** 2015-10-27

**Authors:** Seyed-Ebrahim Kassaian, Sepideh Saroukhani, Farshid Alaeddini, Mojtaba Salarifar, Davide Capodanno, Hamidreza Poorhoseini, Masoumeh Lotfi-Tokaldany, Massoud A Leesar, Hassan Aghajani, Elham Hakki-Kazzazi, Mohammad Alidoosti, Ali-Mohammad Haji-Zeinali, Maryam Saifi, Ebrahim Nematipour

**Affiliations:** 1 *Tehran Heart Center, Tehran University of Medical Sciences, Tehran, Iran.*; 2 *Cardiovascular Department, Ferrarotto Hospital, Catania, Italy.*; 3 *Interventional Cardiology Department, University of Alabama, Birmingham, Alabama, USA.*; 4 *Pediatrics Department, University of Texas Southwestern Medical School, Dallas, Texas, USA.*

**Keywords:** *Patient-specific modeling*, *Prognosis*, *Percutaneous coronary intervention*

## Abstract

**Background: **The aim of the present study was to develop a scoring system for predicting 1-year major adverse cardiac events (MACE), including mortality, target vessel or target lesion revascularization, coronary artery bypass graft surgery, and non-fatal myocardial infarction after percutaneous coronary intervention (PCI).

**Methods: **The data were extracted from a single center PCI registry. The score was created based on the clinical, procedural, and laboratory characteristics of 8206 patients who underwent PCI between April 2004 and October 2009. Consecutive patients undergoing PCI between November 2009 and February 2011 (n= 2875) were included as a validation data set.

**Results:** Diabetes mellitus, increase in the creatinine level, decrease in the left ventricular ejection fraction, presentation with the acute coronary syndrome, number of diseased vessels, primary PCI, PCI on the left anterior descending artery and saphenous vein graft, and stent type and diameter were identified as the predictors of the outcome and used to develop the score (R² = 0.795). The models had adequate goodness of fit (Hosmer-Lemeshow statistic; p value = 0.601) and acceptable ability of discrimination (c-statistics = 0.63). The score categorized the individual patients as low-, moderate-, and high-risk for the occurrence of MACE. The validation of the model indicated a good agreement between the observed and expected risks.

**Conclusion:** An individual risk-scoring system based on both clinical and procedural variables can be used conveniently to predict 1-year MACE after PCI. Risk classification based on this score can assist physicians in decision-making and postprocedural health care.

## Introduction

Percutaneous coronary intervention (PCI) is the most common form of myocardial revascularization.^1^ Several studies have sought to develop prediction models to help physicians predict the clinical outcome after PCI. To date, however, PCI prediction models have predominantly focused on early results such as the in-hospital or 30-day outcome.^2^^, ^^3^ Almost all the currently available models were developed to predict in-hospital mortality and did not account for other major adverse cardiac events (MACE) after PCI and potentially longer-term events. The Mayo Clinic, Cleveland Clinic, and Brigham and Women’s Hospital models are examples that also studied the prediction of other in-hospital adverse events, including myocardial infarction (MI), stroke, and emergency coronary artery bypass graft surgery (CABG).^4^^-^^7^ The number of studies developing prediction models for the long-term detailed outcome of PCI is relatively low.^1^^, ^^8^^-^^11^

There are other limitations to the current risk-adjustment models. First, some of the previous risk models were developed based on risk factors available before the procedure and did not include the anatomical and procedural characteristics that might influence the outcome.^12^^, ^^13^ Second, some of the prediction models were developed for only a specific group of patients (i.e. patients aged > 65 years).^1^ Finally, most of the risk prediction models were developed before the new-generation drug-eluting stent (DES) era and they are not representative of the current practice of PCI.

The aim of the present study was to construct an up-to-date and practical risk-scoring model based on a wide range of clinical, laboratory, and procedural characteristics for the prediction of the 1-year outcome after successful PCI.

## Methods

The data were extracted from a single-center computerized registry of interventional cardiology, which is described in detail elsewhere.^14^ A total of 8206 consecutive patients who underwent PCI between April 2004 and October 2009 and had at least one stent successfully deployed were included in the current analysis. The study protocol was approved by the institutional Review Board. Cardiovascular risk factors were evaluated using the American College of Cardiology-National Cardiovascular Data Registry (ACC-NCDR) definitions as previously reported.^15^ All patients who presented with unstable angina or non-ST elevation MI and underwent PCI were considered as the acute coronary syndrome (ACS). 

In the patients with a successful procedure, clopidogrel (75 mg daily) was prescribed for at least 12 months in the DES group and for at least 1 month in the bare-metal stent (BMS) group. The patients with the DES were discharged with Aspirin (325 mg daily) for at least 3 months, whereas the patients with the BMS were prescribed Aspirin (325 mg daily) for at least 1 month. Aspirin (80 mg daily) was prescribed for an indefinite period after PCI in all the patients. 

The main endpoint of the current study was the occurrence of MACE, defined as death, non-fatal MI, target lesion revascularization (TLR), and target vessel revascularization (TVR), during a 1-year follow-up after discharge. The final model was constructed based on all baseline clinical, laboratory, and diagnostic catheterization data as well as procedural details.

The data are presented as mean ± standard deviation (SD) for the continuous variables, and frequencies and percentages for the categorical variables. A multiple logistic regression model was used to identify the independent predictors of the 1-year outcome. All the evaluated variables were included in the regression model. The area under the receiver operating characteristic (ROC) curves and the Hosmer-Lemeshow test were employed to assess the discriminatory power and the goodness of fit and calibration of the regression model, respectively. 

Our missing data were less than 5%. The left ventricular ejection fraction (LVEF) was the exception, with 9.5% of the relevant data missing. The multiple imputation linear regression method was utilized in order to improve the point estimation of the values in the prediction model. 

**Figure 1:**
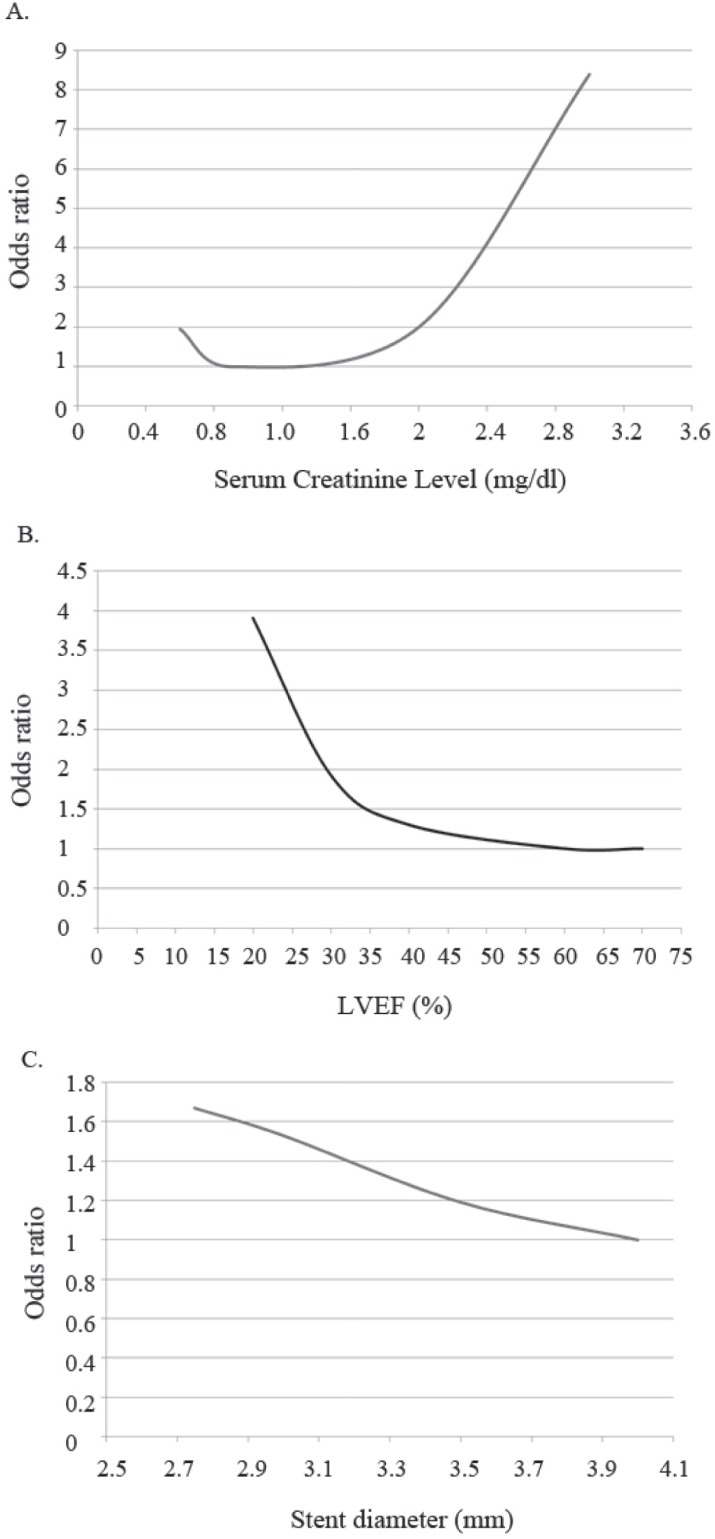
J-shaped relationship between A) serum creatinine level, B) left ventricular ejection fraction (LVEF), and C) stent diameter and 1-year major advers cardiac events

Restricted cubic splines (Devlin T, Weeks B, editors. Spline functions for logistic regression modeling. Proceedings of the 11th Annual SAS Users Group International Conference Cary, NC: SAS Institute, Inc; 1986;646-651.) were used to allow for nonlinear associations between the continuous variables and the endpoint. For each continuous variable, the spline function showed that its effect on the odds ratio of 1-year MACE was approximately linear below a threshold and approximately constant above the same threshold (Figure 1). On the basis of these exploratory analyses, the LVEF was categorized as ≤ 30%, 30% – 40%, and ≥ 40%; serum creatinine level knots were classified at 0.9, 2.0, and 3.0 mg/dL; and stent diameter was categorized as ≤ 3, 3 – 3.5, and > 3.5 mm. The distance between the other categorical variables and their base (reference) category in the regression coefficient units was equal to the size of the coefficient. There was no significant interaction between the variables.

After the finalization of the logistic regression model, the scores were defined by selecting an integer coefficient roughly proportional to the log odds ratio coefficients (i.e. parameter estimates). An integer score was assigned for the real-valued statistical model coefficients of each risk factor significantly associated with the event as follows. The effects from the spline terms were summed in order to reduce the contributions from the continuous variables to 1 number. After that, each variable’s term was divided by a given number and rounded to the nearest integer. Estimates of risk based on the integer score were computed through simple logistic regression. The agreement between the observed and expected risk based on the model was tested on a group of consecutive patients undergoing successful PCI between November 2009 and February 2011 (n = 2875) as a validation data set. Statistical Package for the Social Sciences (SPSS) version 19 (SPSS Inc.; Chicago, Illinois) was used for all the statistical analyses.

## Results

Among the 8206 patients (mean age = 57.1 ± 10.5 years, 70.3% males), in-hospital MACE occurred in 70 (0.9%) patients and 427 (5.2%) cases developed MACE during the follow-up period (after discharge up to 12 months). In other words, a total of 488 (5.9%) patients had at least one adverse event during hospitalization or after discharge during the follow-up. Mortality occurred in 72 (0.9%) patients, among whom 52 (0.6%) cases died due to cardiac events. In addition, 115 (1.4%) patients had nonfatal MI, 75 (0.9%) underwent CABG, 178 (2.2%) underwent TVR, and 78 (1.0%) underwent TLR. Among all the patients, 2532 (30.8%) patients presented with the ACS. All the other baseline clinical, laboratory, and procedural data and their univariate association with the 12-month outcome are depicted in Table 1 and Table 2.

The multiple regression model identified diabetes mellitus, increase in the creatinine level, decrease in the LVEF, presentation with the ACS, number of diseased vessels, primary PCI, and PCI on the left anterior descending artery as the independent predictors of 1-year adverse events. The stent characteristics, including decrease in the stent diameter and deployment of the BMS and the first-generation DES versus the second-generation DES, and PCI on the saphenous vein graft were also associated with the occurrence of 1-year MACE (Table 3). The area under the ROC curve was 0.63 (95% confidence interval: 0.59 – 0.66), indicating the acceptable discriminatory ability of the model. The Hosmer-Lemeshow statistic was not significant (p value = 0.601), demonstrating goodness of fit. 

**Figure 2:**
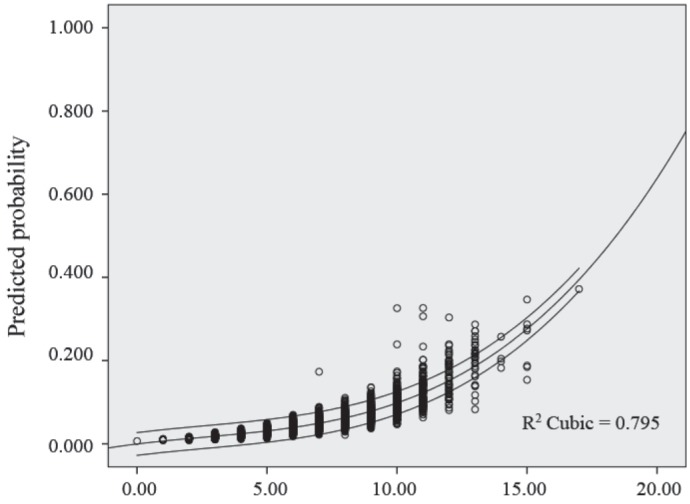
Relation between the risk score and the predicted probability of 1-year major adverse cardiac events (MACE)

The scoring system and the estimated risks, depicted in Table 4, were constructed based on the plots demonstrated in Figure 1 for the continuous variables and the coefficients of each predictive categorical variable in the logistic regression model. The total risk score was calculated by adding up each point related to all the existing predicting factors. Based on the total score, the patients were categorized into three groups of low-, moderate-, and high-risk for the occurrence of 1-year MACE (Table 5). The relation between the risk score and the predicted probability of 1-year MACE was plotted and evaluated using the R-squared statistics (Figure 2). The equation for the log odds ratio of 1-year MACE was “log odds ratio = –3.76 + [0.052 × score] + [0.001 × score³]” and the R-squared was 0.795, indicating a good correlation between the scoring system and the predicted 1-year MACE.

The validation of the model indicated a good agreement between the observed and expected risk in the validation data set (n = 2875) (Table 5). 

**Table 1:** Univariate association between the baseline clinical characteristics and 1-year MACE (n = 8206)*

		Odds Ratio	95% Confidence Interval	P Value
Age (y)	57.1±10.5	1.008	0.999-1.018	0.083
Female	2441 (29.7)	1.014	0.820-1.255	0.895
BMI (kg/m^2^)**	27.6±4.3 (14.5-51.8)	0.992	0.967-1.017	0.506
Hypertension	3689 (45.0)	1.126	0.926-1.370	0.234
Hyperlipidemia	5549 (67.6)	1.007	0.816-1.243	0.948
Diabetes mellitus	2074 (25.3)	1.457	1.182-1.797	< 0.001
Current smoking	1845 (22.6)	0.975	0.771-1.234	0.833
Serum creatinine (mg/dL)***	1.12 (0.1-10.8)	1.240	1.062-1.448	0.006
Creatinine > 2.0 mg/dL	80 (1.0)	2.699	1.380-5.277	0.004
GFR (mL/min/1.73 m²)	71.7±19.4	0.994	0.989-0.999	0.017
Median LVEF (range)	51.9 (10.0-80.0)	0.979	0.970-0.988	< 0.001
LVEF < 45%	2021 (27.2)	1.272	1.018-1.588	0.034
Previous myocardial infarction	3541 (43.2)	1.122	0.922-1.366	0.252
Previous PCI	541 (6.6)	1.711	1.236-2.369	0.001
Previous CABG	292 (3.6)	1.590	1.026-2.462	0.038
Presentation with acute coronary syndrome	2532 (30.8)	1.264	1.031-1.551	0.024
Primary PCI	254 (3.1)	2.911	1.980-4.281	< 0.001
Postprocedural rise in CKMB mass	547 (6.7)	1.787	1.298-2.459	< 0.001
Cardiogenic shock	27 (0.3)	5.732	2.090-15.722	0.001

MACE, Major adverse cardiac events; BMI, Body mass index; GFR, Glomerular filtration rate; LVEF, Left ventricular ejection fraction; PCI, Percutaneous coronary intervention; CABG, Coronary artery bypass graft; CKMB, Creatine kinase-MB

* Data are presented as mean ± SD or n (%).

** BMI is presented as mean (interquartile range).

*** Serum creatinine is presented as median (interquartile range).

**Table 2:** Univariate association between the lesion and procedural characteristics and 1 - Year MACE (n = 8206)*

		Odds Ratio	95% Confidence Interval	P Value
				
Number of diseased vessels				
1VD	3420 (41.7)	1	Ref	
2VD	2955 (36.0)	1.382	(1.094-1.745)	0.007
3VD	1394 (17.0)	1.824	(1.397-2.383)	< 0.001
Lesion				
Mean lesion length (mm)	27.5±15.6	1.011	1.006-1.017	< 0.001
Mean RVD (mm)	3.18±0.80	0.701	0.554-0.887	0.003
Total occlusion	626 (7.6)	1.517	1.160-1.985	0.002
Lesion type C (ACC/AHA classification)	5377 (65.5)	1.187	0.962-1.465	0.110
Bifurcation	740 (9.0)	0.778	0.536-1.131	0.188
Thrombus	232 (2.8)	1.413	0.842-2.372	0.191
In-stent restenosis	157 (1.9)	1.388	0.746-2.583	0.301
Lesion location				
Ostial	617 (7.5)	1.183	0.835-1.675	0.344
Proximal	4793 (58.4)	0.984	0.808-1.199	0.876
LAD	5353 (65.2)	1.177	0.954-1.451	0.128
Proximal part of LAD	2899 (35.3)	1.023	0.835-1.254	0.826
PCI on SVG	115 (1.4)	2.816	1.622-4.891	< 0.001
Stents				
Type of stent				
BMS	3569 (48.8)	2.082	1.458-2.972	< 0.001
First-generation DES**	2438 (33.4)	1.911	1.318-2.772	0.001
Second-generation DES**	1302 (17.8)	1.00	Ref	
Number of stents (per patient)		1.261	1.098-1.449	0.001
1	5997 (73.1)	1.00	Ref	
2	1751 (21.3)	1.312	1.043-1.649	0.020
≥ 3	458 (5.60)	1.720	1.199-2.467	0.003
Number of overlapping stents (per patient)		1.257	0.955-1.654	0.103
0	7483 (91.2)	1.00	Ref	
1	666 (8.1)	1.159	0.825-1.627	0.395
≥ 2	57 (0.7)	2.181	0.930-5.114	0.073
Stent length (mm)	30.3±15.7	1.011	1.006-1.017	< 0.001
Stent diameter (mm)	3.12±0.41	0.641	0.494-0.830	0.001
Stent diameter				
≤ 3 mm	5054 (61.6)	1.767	1.125-2.773	0.013
3.1-3.5 mm	2515 (30.7)	1.409	0.878-2.262	0.155
> 3.5 mm	632 (7.7)	1.00	Ref	
Final inflation pressure (mm Hg)	13.46±2.72	0.989	0.953-1.025	0.531

MACE, Major adverse cardiac events; VD, Vessel disease; RVD, Reference vessel diameter; ACC/AHA, American College of Cardiology/American Heart Association; LAD, Left anterior descending artery; PCI, Percutaneous coronary intervention; SVG, Saphenous vein graft; BMS, Bare-metal stent; DES, Drug-eluting stent

* Data are presented as mean±SD or n (%).

** First-generation DESs include Paclitaxel- and Sirolimus-eluting stents. Second-generation DESs include Everolimus-, Zotarolimus-, and Biolimus-eluting stents.

**Table 3:** Multivariable model for 12-month MACE (death, MI, TVR, TLR, and CABG)

	Odds Ratio	95% Confidence Interval	P Value
Diabetes mellitus	1.294	0.989-1.694	0.060
Creatinine (mg/dL) (linear, per 1-unit increase)	1.288	1.068-1.554	0.008
Spline fit*			0.017
LVEF (%) (linear, per 1-unit increase)	0.985	0.973-0.997	0.014
Spline fit*			< 0.001
Presentation with ACS	1.354	1.041-1.761	0.024
Number of diseased vessels			
1VD	Ref		
2VD	1.319	0.990-1.757	0.059
3VD	1.686	1.196-2.377	0.003
Primary PCI	2.231	1.266-3.933	0.006
PCI on LAD	1.295	0.975-1.721	0.074
Stent version			
BMS	2.460	1.637-3.697	< 0.001
First-generation DES	1.978	1.315-2.977	0.001
Second-generation DES	Ref		
Mean stent diameter (mm) (linear, per 1-unit increase)	0.580	0.418-0.804	0.001
Spline fit*			< 0.001
SVG	2.727	1.303-5.707	0.008

MACE, Major adverse cardiac events; LVEF, Left ventricular ejection fraction; ACS, Acute coronary syndrome; VD, Vessel disease; PCI, Percutaneous coronary intervention; LAD, Left anterior descending artery; BMS, Bare-metal stent; DES, Drug-eluting stent; SVG, Saphenous vein graft

* Serum creatinine level knots at 0.9, 2.0, and 3.0 mg/dL, LVEF knots at 30, 30 - 40, and above 40%, and stent diameter knots at 3, 3 - 3.5, and above 3.5 mm.

**Table 4:** Risk scoring model for the prediction of 1-year MACE

Risk factor	Score
Presentation with acute coronary syndrome	2
Diabetes mellitus	1
Creatinine (mg/dL)	
< 0.6	2
0.6-0.8	1
0.8-1.5	0
1.5-1.8	1
1.8-2.0	2
2-2.4	3
> 2.4	4
Left ventricular ejection fraction (%)	
≤ 25	3
26-30	2
31-45	1
> 45	0
Number of diseased vessels	
1 VD	0
2 VD	1
3 VD	2
Primary PCI	3
PCI on LAD	1
Stent version	
BMS	3
First-generation DES	2
Second-generation DES	0
Mean stent diameter (mm)	
≤ 3	2
3.01 – 3.5	1
> 3.5	0
SVG	3

MACE, Major adverse cardiac events; LVEF, VD, Vessel disease; PCI, Percutaneous coronary intervention; LAD, Left anterior descending artery; BMS, Bare-metal stent; DES, Drug-eluting stent; SVG, Saphenous vein graft

**Table 5:** Risk categories and the frequency of the observed and expected events

	Risk categories		
	Low	Moderate	High
Score intervals	0-6	7-9	≥ 10
Expected events (%)	< 5	5-9	≥ 10
Observed events in developmental set (%)	3.5	5.2	13.9
Observed events in validation set (%)	3.3	4.3	10.3

The equation for the log odds ratio of MACE is “log odds ratio = –3.76 + [0.052 × score] + [0.001 × score³], R² = 0.795, Brier = 0.046.

## Discussion

This study aimed to develop a scoring scale to predict the outcome from discharge up to 1 year, using a wide range of the clinical and procedural characteristics of patients undergoing PCI. In this model, PCI on the saphenous vein graft, primary PCI, and deployment of the BMS in comparison with the first- and second-generation DES were the strongest predictors of 12-month MACE. The model had acceptable discrimination ability and good patient-risk prediction ability (calibration). The predictive performance of the model was validated by reanalysis in a separate group of patients undergoing PCI.

Several scoring systems have been developed to predict in-hospital or at most 30-day mortality or MACE after PCI.^2^^, ^^3^^, ^^5^^-^^7^^, ^^12^ Even more recently, a scoring model was developed to predict 30-day readmission after PCI.^16^ In contrast, there are few scoring models available for the prediction of longer-term outcomes of PCI. Mackenzie et al.^8^ developed a model to predict long-term mortality after PCI, including demographics, comorbidities, severity of the disease, and acuity of the clinical presentation, and reported that age, sex, diabetes mellitus, decreased LVEF, kidney disease, and emergent procedures were the predictors of mortality. However, the authors did not develop a scoring system. The results of another recent model to predict long-term mortality after PCI in elderly adults showed that the variables related to the anatomical severity of the disease and acuity of the presentation were the most powerful predictors of mortality.^1^ Nevertheless, not only were the results only applicable to patients aged ≥ 65 years but also the authors failed to provide a simple scoring system. 

The New Risk Stratification Score (NERS)^17^ categorizes patients into high- and low-risk levels, without the ability to individualize the assessment of patient risk. In addition, it is a complicated score comprising 17 clinical, 33 anatomical, and 4 procedural factors developed for patients with left main coronary artery disease undergoing PCI and is, as such, not applicable to all patients. The Logistic Clinical SYNTAX (synergy between PCI with Taxus and cardiac surgery) score^9^ was developed using the data of seven coronary stent trials, in which adding clinical variables including age, creatinine clearance, and LVEF to the anatomical SYNTAX score resulted in a major improvement of the 1-year predictive ability of all-cause mortality (core model). The study also did a similar analysis after adding six other clinical variables, viz. presentation, body mass index, peripheral vascular disease, diabetes mellitus, previous MI, and smoking, to the core model that had the best univariate associations to the 1-year outcome. However, the extended model showed only a minor incremental benefit compared with the core model. In addition, the findings indicated that neither the core model nor the extended model significantly improved the ability of the SYNTAX score in isolation when used for the prediction of 1-year total MACE. Despite the high similarity between the clinical variables found to be influential on the 1-year outcome in our model and the logistic clinical SYNTAX score, there are some additional differences that should be noted. The impact of the DES generation on the final outcome, which was eminent in our findings, is the principal difference. Most notably, the logistic clinical SYNTAX score was derived from stent trials, each of which had certain inclusion and exclusion criteria and was conducted in different centers with different set-ups. These differences, however minimal, could restrict the application of this score in real-world clinical practice. Another possible explanation for this disparity might be related to the dominant impact of the anatomical SYNTAX, which alleviates the impact of other factors on the long-term outcome. The results of a study indicated that while the angiographically based SYNTAX risk score is proven to triage patients to CABG versus PCI, its use as a predictive tool for patients with extensive coronary disease electing to undergo PCI is limited. Based on the findings, the clinically orientated New York State Risk Score was superior to the SYNTAX score for predicting death and MACE 1 year after PCI (Limaye AM, Patel R, Chandela S, Lee P, Trost B, Karajgikar R, Kovacic JC, Pyo RT, Sweeney J, Suleman J. The Clinically Based New York State Risk Score Is Superior To The Angiographically Based Syntax Risk Score For Predicting Patient Outcomes After PCI. J Am Coll Cardiol 2011;57(14):E1949). It, therefore, appears that the impact of some factors on the long-term outcome may increase when the SYNTAX score is not available.

Some of the previous prediction models were based on merely the clinical variables available before PCI.^12^^, ^^13^ They emphasized this characteristic as an advantage for their analysis believing that risk assessment should be performed before the procedure. In contrast, almost all the procedural variables, including the target vessel, remained as predictors in our analysis, and the type and size of the stents were successfully identified based on the angiographic assessment before PCI. As a result, considering all the aforementioned studies together, it appears that an efficient risk prediction model for patients undergoing PCI should incorporate both clinical and angiographic and procedural factors.

Previous models considered the serum creatinine level and the LVEF as dichotomous variables.^1^^, ^^3^^, ^^8^ In our study, similar to the Mayo Clinic risk score,^12^ the frequency of MACE was high in patients with very high and very low serum creatinine levels, as is depicted in Figure 1. A possible explanation is the low muscle mass or senility seen in patients with very low serum creatinine levels. Based on this finding, we considered the serum creatinine level, LVEF, and stent diameter as J-shaped plots. Consequently, we calculated and utilized the gradient of the odds ratios with minor changes in the values of serum creatinine, LVEF, and stent diameter to score the values of these variables more accurately in the model. 

In our study, the deployment of the first-generation DES was associated with approximately a twofold higher risk of the occurrence of MACE in comparison with the new-generation DES. To our knowledge, our study is one of the few studies conducted after the development of the new generations of the DES. The more recent models^1^^, ^^9^ were also developed after the presence of the new-generation DES. However, the relevant studies did not include and compare the different types of the DES in their analyses. These findings highlight the advantageous feature of considering the types of the DES in our study and the significant impact of the procedural characteristics on the final outcome of PCI. 

Cardiogenic shock has been consistently shown to be a powerful predictor of in-hospital mortality.^3^^, ^^9^^, ^^12^^, ^^13^^, ^^18^^-^^20^ Nonetheless, its impact on the long-term outcome has been demonstrated to be less predominant.^1^ In our study, despite the significant effect of cardiogenic shock, shown in the univariate analysis (odds ratio: 5.732), it did not remain as a predictor in the multivariable model. The reason may lie in its close relation with primary PCI, which was a strong predictor of MACE in the final model.

There are some other advantageous aspects that enhance the clinical implication of the present risk score. To begin with, in contrast to most of the previous models,^1^^, ^^8^ the present risk score can be applied to the risk stratification of total MACE 12 months after PCI. Furthermore, some of the previous studies failed to provide a scoring system to simplify risk prediction.^1^^, ^^8^ An additive integer-scoring tool enables physicians to quickly sum the coefficients for a numerical ranking of patient risk and helps them decide on a treatment plan and selection of suitable devices. 

This study has a number of limitations. Although our center is a high-volume tertiary care referral center with a real-world PCI registry, it still has the shortcoming of being a single-center registry, which may weaken the generalizability of our model in comparison with multi-center or national models. For instance, age was identified as a predictor of the PCI outcome in most of the previous models^2^^, ^^8^^-^^10^^, ^^12^ but not in our study. One possible explanation is that the mean age of our patients was almost 10 years younger than the mean age of the patients included in the previous studies. This point might undermine the ability of our model to be generalized to patients aged > 60 years. The other inadequacies of the present study are related to the lack of detailed data on some variables. For instance, despite the prescription of the standard dual antiplatelet therapy for all the patients during the follow-up, accurate information on the duration of this therapy in the follow-up period was not available in our data. In addition, some potential risk factors such as history of peripheral vascular disease or chronic obstructive pulmonary disease, presentation with heart failure, and anatomical SYNTAX score are not available routinely in our registry, which may influence the performance of the model. This point also precluded a comparison between our score and the other available risk scores such as the clinical SYNTAX score. Another salient point is that primary PCI was not performed 24/7 in our center during the study period and a many MI patients received thrombolytic therapy. Therefore, the number of the patients with ST-segment elevation myocardial infarction (STEMI) and cardiogenic shock who underwent primary PCI was lower than the real number of the patients who were admitted with STEMI and cardiogenic shock. Finally, our model was internally validated in this analysis and the generalizability of the model requires external validation by future studies. 

## Conclusion

The present risk score was developed for the 1-year outcome risk stratification and applies to patients undergoing PCI at any age, in the new-generation DES era. This score confers the ability to assess risk for each patient based on the related demographic, clinical, angiographic, and procedural characteristics. The inclusion of the procedural variables in the present study can enhance its ability to help clinicians select suitable devices and stents by estimating the prognosis with considerable accuracy both initially and during the follow-up after PCI. Future studies can focus on additional clinical and procedural variables not routinely collected in registries that may improve the performance of the prediction model.

## Notes:

***This paper should be cited as:*** Kassaian SE, Saroukhani S, Alaeddini F, Salarifar M, Capodanno D, Poorhoseini H, Lotfi-Tokaldany M, Leesar MA, Aghajani H, Hakki-Kazzazi E, Alidoosti M, Haji-Zeinali AM, Saifi M, Nematipour E. A Risk-Scoring Model to Predict One-year Major Adverse Cardiac Events after Percutaneous Coronary Intervention. J Teh Univ Heart Ctr 2015;10(4):167-175.
